# Supplementary material for: Sublethal Radiation Affects Antigen Processing and Presentation Genes to Enhance Immunogenicity of Cancer Cells
Source: Int J Mol Sci. 2020 Apr 7;21(7):2573. doi: 10.3390/ijms21072573 (PMC7178186; doi:10.3390/ijms21072573)
Supplement: Supplementary file 1 [file ijms-21-02573-s001.zip › Supplementary_materials.pdf]

Supplementary materials

Punnanitinont et al., 2020, on effect of radiation on cancer cell-lines.

1. Figure S1. Effect of radiation on cell viability after four days.
2. Figure S2. Effect of radiation on cell surface protein measurement by flow cytometry.
3. Figure S3. Effect of radiation on cell size measurement by flow cytometry.
4. Figure S4. Effect of radiation on expression of genes encoding cancer/testis antigens (CTAs).
5. Table S1. Results of differential gene expression analyses applied to identify effect of radiation.
6. Table S2. Genes whose expression was down-regulated by radiation in all cell-lines.

Figure S1. *Effect of radiation on cell viability.* Sub-confluent adherent cultures of indicated cell-lines that had been grown in parallel to same cell density were treated with one dose of 0, 7.5, or 15 Gy X-rays. Viability of adherent cells after four days was assessed from 7-amino-actinomycin D staining in fluorescence flow cytometry. Mean and its standard error for viability measurements in three independent experiments are plotted.

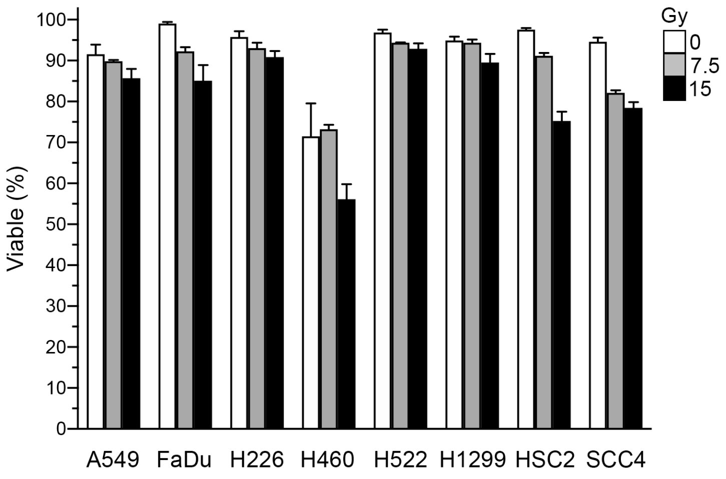

Figure S2. *Effect of radiation on cell surface protein measurement by flow cytometry.* Sub-confluent adherent cultures of indicated cell-lines that had been grown in parallel to same cell density were treated with one dose of 0, 7.5, or 15 Gy X-rays. After four days, adherent cells were collected by scraping and examined by direct fluorescence flow cytometry for cell surface expression of CD23, calreticulin (*CALR*), and mucin 1 (*MUC1*) proteins based on the binding of fluorophore-conjugated mouse IgG monoclonal antibodies (mAb) against them. Binding of IgG1 fraction of normal mouse serum (*mIgG1*) and of an anti-NY-ESO-1 mIgG1 mAb was indirectly assessed with a fluorophore-conjugated rat anti-mIgG1 mAb. Geometric mean fluorescence intensity (*GMFI*) values of viable cells based on *7*-amino-actinomycin D staining was calculated from flow cytometry data. Mean and its standard error for GMFI determinations in three independent experiments are plotted.

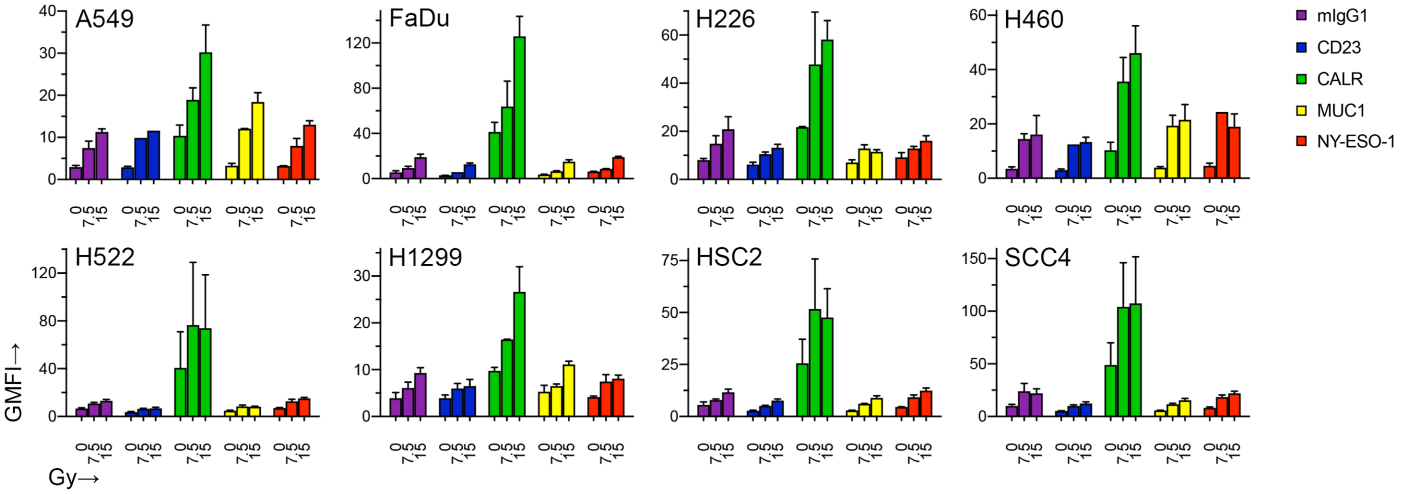

Figure S3. *Effect of radiation on cell size measurement by flow cytometry.* Sub-confluent adherent cultures of indicated cell-lines that had been grown in parallel to same cell density were treated with one dose of 0 or 15 Gy X-rays. After four days, adherent cells were collected by scraping and examined by flow cytometry. Representative histograms of forward scatter of viable cells identified by *7*-amino-actinomycin D staining are shown.

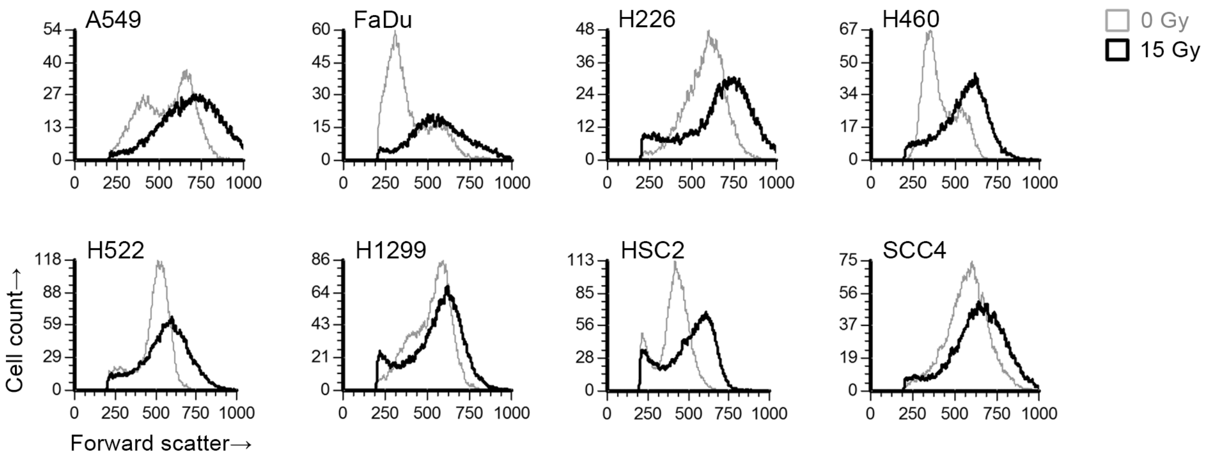

Figure S4. *Effect of radiation on expression of genes encoding cancer/testis antigens (CTAs).* Heatmap shows effect of 15 Gy radiation, calculated as log_2 fold-change compared to untreated cells, on expression in indicated cell-lines of 105 CTA genes whose expression was detected in at least one cell-line. Genes for which there was no effect are in grey (nominal P ≥ 0.05 in likelihood ratio test in edgeR). Those with $P < 0.05$ are colored as per the displayed scale. Genes whose expression was considered too low were not examined and are in white. Sub-confluent adherent cultures of cell-lines that had been grown in parallel to same cell density were treated with one dose of 0 or 15 Gy X-rays in three independent experiments. After four days, adherent cells were examined for gene expression by RNA sequencing.

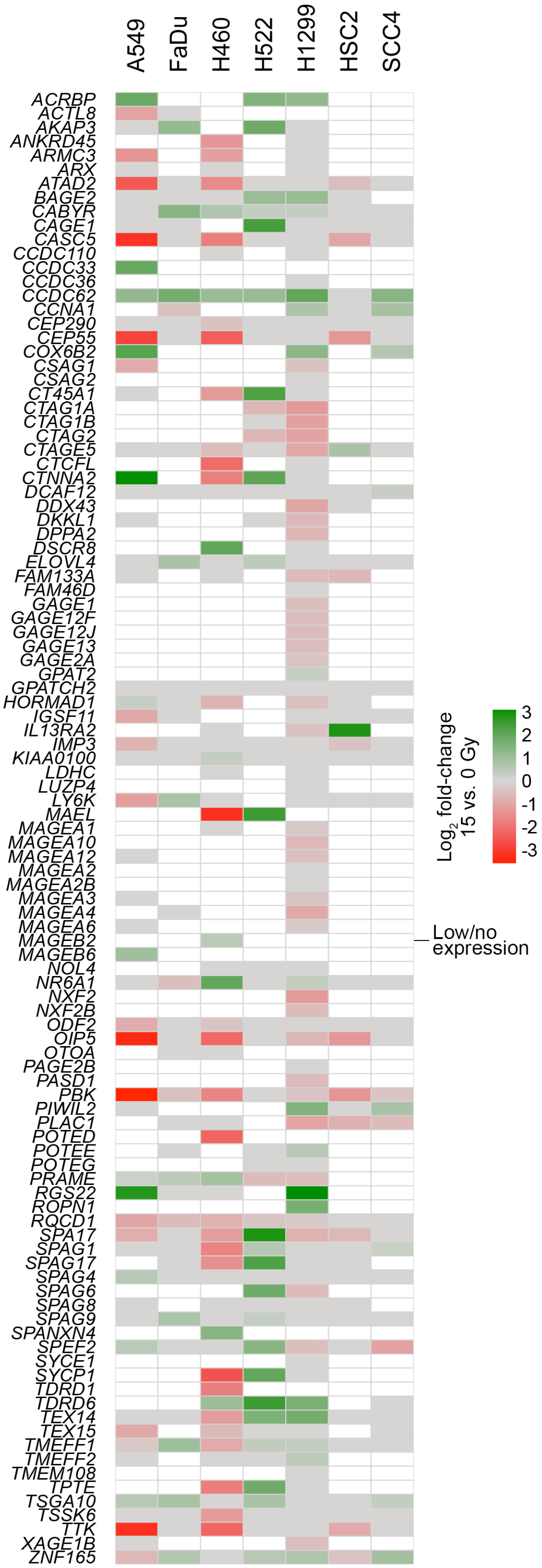

Table S1. Results of differential gene expression analyses applied to identify effect of radiation.

File *Table S1.xlsx* (Microsoft® Excel™ document, 9.4 MB).

Table S2. Genes whose expression was down-regulated by radiation in all cell-lines[a].

Gene	*Description*	*Log_2 fold-change*	*P*
ABCB10	ATP-binding cassette, sub-family B (MDR/TAP), member 10	-1.1–-0.4	3.0E-15–1.5E-02
AK2	Adenylate kinase 2	-0.8–-0.2	4.6E-23–4.6E-02
AL662801.1	C6orf47 antisense RNA 1	-0.7–-0.3	4.3E-12–9.2E-03
ALYREF	Aly/REF export factor	-1.8–-0.2	1.9E-74–3.5E-02
ATP6V1G2-DDX39B	ATP6V1G2-DDX39B readthrough (NMD candidate)	-0.7–-0.3	1.1E-12–4.1E-02
BCL7C	B-cell CLL/lymphoma 7C	-0.9–-0.3	6.5E-11–4.7E-02
BTF3	Basic transcription factor 3	-0.6–-0.2	1.7E-08–2.4E-02
C12orf10	Chromosome 12 open reading frame 10	-0.8–-0.4	5.1E-07–3.2E-02
C17orf75	Chromosome 17 open reading frame 75	-0.7–-0.2	1.9E-05–2.2E-02
C8orf82	Chromosome 8 open reading frame 82	-1.0–-0.4	1.7E-09–4.9E-02
CDCA7	Cell division cycle associated 7	-1.5–-0.3	2.4E-60–3.9E-02
CDKN2C	Cyclin-dependent kinase inhibitor 2C (p18, inhibits CDK4)	-1.8–-0.5	1.3E-37–2.3E-02
COX20	COX20 cytochrome c oxidase assembly factor	-1.1–-0.3	1.1E-17–2.1E-02
CYC1	Cytochrome c-1	-1.0–-0.3	1.7E-30–4.0E-02
DCPS	Decapping enzyme, scavenger	-1.5–-0.5	1.0E-21–2.5E-02
DHPS	Deoxyhypusine synthase	-0.9–-0.3	6.6E-19–2.3E-02
ECI2	Enoyl-CoA delta isomerase 2	-1.0–-0.4	7.1E-08–7.9E-03
EEF2KMT	Eukaryotic elongation factor 2 lysine methyltransferase	-1.3–-0.4	1.0E-16–4.2E-02
ENO1	Enolase 1, (alpha)	-0.9–-0.2	3.1E-09–8.2E-03
ESD	Esterase D	-0.9–-0.4	2.0E-09–4.0E-02
FAM20B	Family with sequence similarity 20, member B	-0.9–-0.3	6.2E-22–7.3E-03
FH	Fumarate hydratase	-0.8–-0.4	4.2E-23–2.0E-02
H2AFV	H2A histone family, member V	-1.2–-0.2	9.4E-35–9.0E-03
H2BFS	H2B histone family, member S (pseudogene)	-2.2–-0.8	2.2E-13–1.6E-02
H3F3B	H3 histone, family 3B (H3.3B)	-0.9–-0.3	5.5E-13–2.8E-02
HIST1H1B	Histone cluster 1, H1b	-4.2–-0.9	1.7E-219–4.8E-09
HIST1H1C	Histone cluster 1, H1c	-2.3–-1.3	4.6E-75–9.3E-12
HIST1H1D	Histone cluster 1, H1d	-3.8–-1.1	5.7E-142–1.1E-05
HIST1H1E	Histone cluster 1, H1e	-2.7–-1.1	2.6E-74–5.1E-06
HIST1H2AB	Histone cluster 1, H2ab	-3.9–-0.8	1.1E-140–2.3E-04
HIST1H2AC	Histone cluster 1, H2ac	-1.6–-0.2	6.8E-40–4.0E-02
HIST1H2AH	Histone cluster 1, H2ah	-4.0–-1.0	7.4E-166–3.9E-09
HIST1H2AI	Histone cluster 1, H2ai	-3.9–-1.1	3.8E-119–4.1E-07
HIST1H2AK	Histone cluster 1, H2ak	-3.2–-0.9	4.5E-82–3.8E-05
HIST1H2AL	Histone cluster 1, H2al	-4.2–-1.1	2.1E-86–1.7E-04
HIST1H2AM	Histone cluster 1, H2am	-3.5–-1.0	1.1E-133–2.3E-05
HIST1H2BB	Histone cluster 1, H2bb	-3.1–-1.0	9.2E-151–1.4E-08
HIST1H2BC	Histone cluster 1, H2bc	-2.6–-0.8	2.4E-118–4.2E-04
HIST1H2BD	Histone cluster 1, H2bd	-2.7–-0.7	2.0E-121–1.0E-06
HIST1H2BJ	Histone cluster 1, H2bj	-3.5–-1.1	4.9E-109–6.1E-08
HIST1H2BK	Histone cluster 1, H2bk	-2.6–-0.7	1.8E-72–5.1E-08
HIST1H2BL	Histone cluster 1, H2bl	-3.9–-1.0	1.3E-154–5.4E-05
HIST1H2BN	Histone cluster 1, H2bn	-3.2–-0.7	5.0E-145–7.0E-07
HIST1H2BO	Histone cluster 1, H2bo	-3.9–-0.8	1.3E-140–3.5E-05
HIST1H3A	Histone cluster 1, H3a	-3.1–-1.2	1.3E-103–4.7E-05
HIST1H3B	Histone cluster 1, H3b	-4.0–-1.4	1.8E-166–1.9E-13
HIST1H3H	Histone cluster 1, H3h	-3.9–-1.0	5.5E-212–2.0E-08
HIST1H3J	Histone cluster 1, H3j	-3.8–-0.9	1.4E-142–5.3E-06
HIST1H4A	Histone cluster 1, H4a	-3.7–-1.1	1.7E-104–4.5E-05

HIST1H4B	Histone cluster 1, H4b	-3.0–-1.4	3.4E-89–3.7E-09
HIST1H4C	Histone cluster 1, H4c	-2.7–-1.1	1.4E-67–3.7E-15
HIST1H4D	Histone cluster 1, H4d	-3.4–-0.7	2.0E-78–6.5E-05
HIST1H4E	Histone cluster 1, H4e	-2.0–-1.1	1.1E-59–2.4E-11
HIST1H4H	Histone cluster 1, H4h	-2.9–-1.2	3.6E-96–4.6E-11
HIST1H4J	Histone cluster 1, H4j	-3.2–-0.8	1.0E-42–7.2E-04
HIST1H4K	Histone cluster 1, H4k	-2.5–-0.7	1.9E-20–3.8E-04
HIST2H2AA3	Histone cluster 2, H2aa3	-3.0–-0.8	1.7E-39–1.6E-02
HIST2H2AA4	Histone cluster 2, H2aa4	-2.7–-1.1	5.3E-56–2.3E-05
HIST2H2AB	Histone cluster 2, H2ab	-3.8–-1.0	1.2E-163–2.7E-07
HIST2H2AC	Histone cluster 2, H2ac	-2.4–-0.9	2.3E-67–2.1E-03
HIST2H2BD	Histone cluster 2, H2bd (pseudogene)	-3.3–-0.8	2.1E-68–2.3E-03
HIST2H2BE	Histone cluster 2, H2be	-2.4–-0.8	2.2E-78–1.3E-05
HIST2H2BF	Histone cluster 2, H2bf	-3.3–-0.5	1.9E-161–4.7E-02
HIST2H3A	Histone cluster 2, H3a	-4.0–-0.9	3.3E-106–1.4E-03
HIST2H3C	Histone cluster 2, H3c	-4.4–-0.8	3.2E-78–6.7E-04
HIST4H4	Histone cluster 4, H4	-1.6–-0.8	5.8E-37–7.5E-06
HMGB1	High mobility group box 1	-1.7–-0.4	1.4E-63–3.5E-03
HMGB1P5	High mobility group box 1 pseudogene 5	-2.0–-0.5	5.7E-66–3.2E-03
HMGN2	High mobility group nucleosomal binding domain 2	-1.7–-0.4	1.3E-61–6.6E-03
HMGN2P3	HMGN2 pseudogene 3	-1.8–-0.5	2.2E-34–3.5E-02
LBR	Lamin B receptor	-1.8–-0.4	1.9E-98–8.2E-03
LMNB1	Lamin B1	-2.8–-0.3	7.9E-143–1.4E-02
LSM2	LSM2 homolog, U6 small nuclear RNA associated	-1.5–-0.4	1.2E-29–5.4E-03
MCRS1	Microspherule protein 1	-0.8–-0.3	2.8E-19–8.6E-03
MRPL3	Mitochondrial ribosomal protein L3	-1.1–-0.3	2.1E-23–5.0E-02
MTFP1	Mitochondrial fission process 1	-1.4–-0.5	3.5E-31–5.1E-03
NDC1	NDC1 transmembrane nucleoporin	-1.8–-0.3	2.3E-92–3.3E-02
NOL7	Nucleolar protein 7, 27kDa	-0.9–-0.3	3.0E-17–2.8E-02
NPM3	Nucleophosmin/nucleoplasmin 3	-1.5–-0.3	2.5E-38–3.9E-03
NUCKS1	Nuclear casein kinase and cyclin-dependent kinase substrate 1	-1.1–-0.4	2.0E-20–1.5E-03
PIR	Pirin (iron-binding nuclear protein)	-1.2–-0.4	5.5E-13–1.1E-02
POLD2	Polymerase (DNA directed), delta 2, accessory subunit	-1.2–-0.2	1.7E-36–3.1E-02
PPIL1	Peptidylprolyl isomerase (cyclophilin)-like 1	-1.0–-0.3	4.9E-24–3.4E-02
PPP1CA	Protein phosphatase 1, catalytic subunit, alpha isozyme	-0.8–-0.2	3.8E-18–3.5E-02
PRMT1	Protein arginine methyltransferase 1	-1.2–-0.2	2.4E-41–4.3E-02
PSMA1	Proteasome (prosome, macropain) subunit, alpha type, 1	-0.6–-0.3	8.1E-08–3.5E-02
PTMA	Prothymosin, alpha	-1.5–-0.5	3.3E-45–8.4E-03
RBMX	RNA binding motif protein, X-linked	-0.9–-0.4	4.3E-22–1.3E-02
RCC1	Regulator of chromosome condensation 1	-0.8–-0.2	2.5E-19–2.3E-02
SET	SET nuclear proto-oncogene	-1.2–-0.2	1.2E-27–3.8E-02
SLC25A15	Solute carrier family 25, member 15	-1.0–-0.4	4.3E-16–3.7E-02
SLC25A5	Solute carrier family 25, member 5	-1.1–-0.4	1.3E-22–1.3E-02
SRSF2	Serine/arginine-rich splicing factor 2	-1.1–-0.3	2.6E-37–2.7E-02
SSRP1	Structure specific recognition protein 1	-1.6–-0.4	3.7E-60–6.7E-03
STAG1	Stromal antigen 1	-1.0–-0.4	2.5E-20–2.2E-02
SUMO3	Small ubiquitin-like modifier 3	-0.8–-0.3	3.6E-12–6.1E-03
TAF9B	TAF9B RNA polymerase II, TBP-associated factor, 31kDa	-1.2–-0.6	9.8E-25–1.3E-04
TRMT112	TRNA methyltransferase 11-2 homolog (S. cerevisiae)	-1.0–-0.3	1.8E-27–3.1E-02
UQCC3	Ubiquinol-cytochrome c reductase complex assembly factor 3	-1.2–-0.4	1.1E-27–4.1E-02
WDR34	WD repeat domain 34	-1.1–-0.5	1.2E-34–9.1E-03
XRCC6	X-ray repair complementing defective repair in Chinese hamster cells 6	-1.0–-0.3	5.9E-24–1.1E-02
ZNRD1	Zinc ribbon domain containing 1	-0.8–-0.4	2.0E-08–4.5E-02

[a]Gene expression differences between three pairs of 15 Gy-irradiated and non-irradiated cells of seven cell-lines was analyzed with the likelihood ratio test in edgeR software. Genes down-regulated by radiation with nominal $P \leq 0.05$ in all cell-lines are listed along with ranges of $\log_2$ fold-change (15 Gy vs. 0 Gy) and P values among the cell-lines.
